# Resveratrol activates CD8^+^ T cells through IL-18 bystander activation in lung adenocarcinoma

**DOI:** 10.3389/fphar.2022.1031438

**Published:** 2022-10-20

**Authors:** Wei Zhang, Ruohao Zhang, Zhiguang Chang, Xiaobo Wang

**Affiliations:** ^1^ Emergency and Disaster Medical Center, The Seventh Affiliated Hospital, Sun Yat-Sen University, Shenzhen, China; ^2^ School of Medicine, Sun Yat-sen University, Shenzhen, China; ^3^ Edmond H. Fischer Translational Medical Research Laboratory, Scientific Research Center, The Seventh Affiliated Hospital, Sun Yat-sen University, Shenzhen, China; ^4^ Department of Hematology, The Seventh Affiliated Hospital, Sun Yat-sen University, Shenzhen, China

**Keywords:** resveratrol (CID: 445154), CD8+T cell, IL-18, bystander activation of T cells, tumor-associated macrophage, lung adenocarcinoma

## Abstract

Resveratrol, a natural product, has demonstrated anti-tumor effects in various kinds of tumor types, including colon, breast, and pancreatic cancers. Most research has focused on the inhibitory effects of resveratrol on tumor cells themselves rather than resveratrol’s effects on tumor immunology. In this study, we found that resveratrol inhibited the growth of lung adenocarcinoma in a subcutaneous tumor model by using the β-cyclodextrin-resveratrol inclusion complex. After resveratrol treatment, the proportion of M2-like tumor-associated macrophages (TAMs) was reduced and tumor-infiltrating CD8T cells showed significantly increased activation. The results of co-culture and antibody neutralization experiments suggested that macrophage-derived IL-18 may be a key cytokine in the resveratrol anti-tumor effect of CD8T cell activation. The results of this study demonstrate a novel view of the mechanisms of resveratrol tumor suppression. This natural product could reprogram TAMs and CD8T effector cells for tumor treatment.

## Introduction

Natural products with free radical scavenging activity have shown potential for tumor treatment and prevention. Polyphenols include a large class of antioxidants, including flavonoids, anthocyanins, phenolic acids, lignans, and stilbenes. These compounds are all derived from phenylalanine and contain aromatic rings with active hydroxyl groups (Dixon, 2001). Resveratrol, a member of the stilbene subclass, is a type of defensive molecule called phytoalexin, which can prevent infection and damage caused by exposure to ultraviolet (UV) irradiation. Resveratrol and its analogs have been identified in several edible natural products such as grapevine, peanut (*Arachis* spp.) (Sanders et al., 2000), berries (Rimando et al., 2004), and rhubarb (*Rheum* spp.) (Matsuda et al., 2001).

As a health care product, resveratrol has been widely confirmed to be safe. Moreover, substantial evidence has shown that resveratrol and its analogs exert cardiovascular protective effects by anti-inflammatory activities and scavenging free radicals generated by oxidation (Ashraf et al., 2015). Recently, resveratrol has also been widely assessed in tumor growth inhibition studies. The anti-tumor effects of resveratrol were first reported in 1997 (Jang et al., 1997). Since then, the antioxidative, anti-inflammatory, anti-proliferative, and anti-angiogenic effects of resveratrol have been extensively studied. Recent reports have shown that resveratrol inhibits the proliferation of several cancers, including colon cancer, breast cancer, pancreatic cancer, prostate cancer, ovarian cancer, and lymphoma through different pathways (Ulrich et al., 2006; Bjorklund et al., 2011; Frazzi et al., 2013).

Resveratrol is considered to directly inhibit cell proliferation by inducing tumor cell senescence (Yang et al., 2013), modulating sub-pools of sphingolipids (Signorelli et al., 2009; Shin et al., 2012), or inducing apoptosis (Aggarwal et al., 2004; Savio et al., 2022). Mechanistically, resveratrol can inhibit PKC (Atten et al., 2001) or MEK1/2-ERK1/2-c-Jun (Aquilano et al., 2009) activity. Reports have also demonstrated that resveratrol can also exert anti-tumor effects by affecting other cells, including immune cells (Chhabra et al., 2021; Mu and Najafi, 2021), stromal cells (Guo et al., 2021) and cancer stem-like cells (Pradhan et al., 2021) in tumors.

Lung cancer is the leading cause of cancer deaths (18.0% of total cancer deaths) (Sung et al., 2021). Lung adenocarcinoma accounts for about 40% of the total number of lung patients. At present, chemotherapy for lung adenocarcinoma has a high effective rate and a significantly improved survival rate. The incidence of EGFR, ALK, and other sensitive mutations in lung adenocarcinoma is also significantly higher than that in other lung cancers. However, the 5-year survival rate for stage II lung adenocarcinoma is only 40–50%. The 5-year survival rates for stage 3A and 3B disease are 25%–30%, and 5%–17%, respectively. The prolongation of the survival time of patients with lung adenocarcinoma by using new tumor treatment methods is a key question for lung adenocarcinoma research. Effective drugs with fewer side effects are needed. In this study, we evaluated the potential of resveratrol for the treatment of lung adenocarcinoma by investigating the changes in the activation of innate and adaptive immune cells during tumor treatment.

## Materials and methods

### Cell lines

Lewis lung carcinoma (LLC) cells were obtained from American Type Culture Collection (ATCC). The LLC cell line was established from the lung of a C57BL mouse bearing a tumor resulting from the implantation of primary LLC. The cell line is highly tumorigenic and is primarily used to model metastasis and evaluate the efficacy of chemotherapeutic agents *in vivo* (Sakai et al., 2006). THP-1 cells were also obtained from ATCC. THP1 is a human leukemia monocytic cell line that has been extensively used to study monocyte/macrophage function (Chanput et al., 2014). Finally, the RAW 264.7 murine macrophage cell line was obtained from Procell Life Science & Technology Co., Ltd., LLC. The Raw264.7 cells were maintained in DMEM+10% FBS+1% P/S/ The THP1 cells were maintained in RPMI-1640 + 10% FBS+1% P/S. All cell lines were cultured in 5% CO_2_ at 37°C.

### Resveratrol-βcd preparation

β-Cyclodextrin-resveratrol inclusion complex was prepared as previously described, with some modifications (Venuti et al., 2014). Sulfobutylether-β-cyclodextrin (Captisol, βcd, Shanghai Chineway Pharmaceutical Tech. Co., Ltd.) was solubilized in water at room temperature and added to an amount of resveratrol (MCE, HY-16561) exceeding its intrinsic solubility. The mix was sonicated in a water bath and then stirred. The suspension was filtered through 0.22 μm filters and freeze-dried. The stoichiometry of the complex was confirmed using Job’s plotting method. A maximum value at R = 0.5 and a symmetrical shape indicated a 1:1 complex. The water solubilities of the resveratrol and the resveratrol-βcd complex were determined by suspending excess drug amounts in 10 ml water and stirring at 25 ± 0.1°C for 24 h. The suspensions were then filtered through 0.22 μm filters and analyzed by UV–vis spectroscopy at 305 nm.

### CCK8 assay

CCK8 (Solarbio, CA1210) was used for the analysis of cell proliferation according to the manufacturer’s instructions. Briefly, cells were seeded in 96-well plates and treated with different drugs, then placed in a 37°C, 5% CO_2_ incubator for pre-culture. For the analysis of CD8T cell proliferation, the upper layer of co-culture was collected and centrifuged at 500 *g* for 5 min to collect the cell pellets, which were then resuspended in 100 μl of culture medium and added to a 96-well plate. Next, 10 µl of CCK-8 solution was added to each well. The culture plates were incubated at 37°C for 60 min. The absorbance at 450 nm was measured using a microplate reader.

### Development and treatment of the lung adenocarcinoma subcutaneous tumor model

Six to eight-week-old C57BL/6J mice were purchased from GemPharmatech Co., Ltd. The study protocols were approved by the Institutional Animal Care and Use Committee of Sun Yat-Sen University. To construct the tumor subcutaneous model, each mouse was shaved and subsequently subcutaneously inoculated with 10^6^ LLC cancer cells. After the tumor volume reached 300 mm^3^, 5 mg/kg resveratrol-βcd was injected peritumorally every 2 days. The resveratrol-βcd injection site was sterilized with 70% alcohol, the injection needle was inserted obliquely, and the drug was injected 3–5 mm under the skin. Syringe draw-back was avoided and the volume for each injection did not exceed 50 μl to avoid necrosis. The mice were sacrificed when the tumor volumes reached approximately 2000 mm^3^ (tumor volume = 1/2 × a × b^2^; a = length and b = width). The tumor tissues were collected for subsequent experiments.

### Flow cytometry

The following antibodies were used for the analysis of tumor infiltration by immune cells: CD45 -eFluor 450 (eBioscience 48-0451-82), CD11b-APC cy7 (eBioscience 47-0112-82), F4/80-PE (eBioscience 12-4801-82), CD8-PE cy7 (eBioscience 25-0081-82), CD4-FITC (eBioscience 11-0042-85), IFNgama-APC (eBioscience 17-7311-82), CD206-APC (eBioscience 17-2061-82). Single-cell suspensions were prepared from the tumor tissues after homogenization or from culture suspension cells. For staining, 100 μl of single-cell suspensions were incubated with antibodies for 30 min. Individual single-color controls were prepared for compensation adjustment. After staining, the samples were washed twice with PBS and resuspended in 600 μl PBS. Flow cytometry data were acquired using a CytoFLEX LX system (Beckman Coulter). FlowJo version 10.0 was used for data analysis. The absolute numbers of CD8^+^ T cell or macrophage numbers per gram of tumor were the corresponding cell counts from 60 μl of cell suspension (10% of total volume) divided by the tumor weight. The gating strategy is described in each figure’s caption. An example of the gating strategy is given in Supplementary Figure S1.

### Cell apoptosis assay

The Annexin V-FITC/PI Kit (Yeasen Biotechnology, Shanghai) was used for the cell apoptosis assay according to the manufacturer’s instructions. Briefly, treated LLC cells were digested with EDTA-free trypsin and harvested by centrifugation at 200 *g* at 4°C for 5 min. Trypsin digestion time was controlled to prevent false positives. The cells were washed with pre-cooled PBS twice at 200 *g* at 4°C for 5 min. The cells were then resuspended in 100 μl of 1× Binding Buffer. Next, 5 μl Annexin V-FITC and 10 μl PI Staining Solution were added. The cells were stained in the dark and at room temperature for 15 min. Next, 400 μl of 1×Binding Buffer was added to the samples and cell apoptosis was detected by flow cytometry within 1 h.

A commercial TUNEL Apoptosis Detection Kit (Alexa Fluor 488) (Yeasen Biotechnology, Shanghai) was used for the analysis of subcutaneous tumor apoptosis. The tumor tissues were embedded in paraffin and sectioned. The sections were immersed in xylene for 5 min twice at room temperature to completely remove the paraffin. The sections were then soaked in 100% ethanol for 5 min at room temperature and then in a 90, 80, and 70% ethanol gradient for 3 min each. The sections were then gently rinsed with PBS. Next, 100 μl of Proteinase K solution at 20 μg/ml was added to each sample and incubated at room temperature for 20 min. The samples were then rinsed three times with PBS solution and the excess liquid was gently removed. Next, 100 μl of 1× Equilibration Buffer was added to each sample to completely equilibrate the sample area and then incubated for 30 min. After removal of the Equilibration Buffer, 50 μl of TdT Incubation Buffer was added to the sample and incubated at 37°C for 60 min in the dark. The samples were then washed three times with PBS and the slides were immersed in a staining tank containing DAPI solution (2 μg/ml, freshly prepared and diluted with PBS) in the dark for 5 min. The samples were then washed three times with deionized water, and 100 μl PBS was added to the sample area to keep the samples moist. The samples were immediately analyzed under a fluorescence microscope.

### Cell senescence analysis

At 1–7 days after resveratrol-βcd treatment, the cells were digested with EDTA-free trypsin and harvested by centrifugation at 200 *g* at 4°C for 5 min. Next, they were washed with PBS, and flow cytometry was performed using p16 as a marker of cell senescence. After 30 min of incubation with p16INK4a antibody (ThermoFisher, PA5-119712), the cells were stained with Alexa Fluor 488-conjugated goat anti-rabbit IgG secondary antibody at a 1:2000 dilution for 30 min. Flow cytometry data were acquired on a CytoFLEX LX system (Beckman Coulter). FlowJo version 10.0 was used for data analysis. For β-gal analysis, a commercial Senescence β-Galactosidase Staining Kit (Beyotime Biotechnology) was used according to the manufacturer’s instructions. Cells in 24-well culture dishes were washed once with PBS. Next, 250 μl β-galactosidase staining fixation solution was added and the samples were incubated for 15 min at room temperature. After fixation, the cells were washed three times with PBS for 3 min each. Next, 250 μl of staining working solution was added to each well and the plates were incubated overnight at 37°C. The plates were sealed with Parafilm or plastic wrap to prevent evaporation. The results were observed and counted by light microscopy.

### Macrophage polarization and CD8T cell separation

Macrophage polarization was performed as previously described (Smith et al., 2015). Briefly, 10 ng/ml PMA was first used to activate THP1 cells for 24 h, making the cells adhere to the plate wall as M0 macrophages. The M0 macrophages were further activated with LPS or IL-4+IL-13 to polarize to M1 or M2 for subsequent experiments. The polarized macrophages were washed three times with serum-free culture medium and then co-cultured with separated CD8T cells. In some co-culture experiments, an anti-hIL18 antibody (InvivoGen) was added to neutralize IL-18 in the culture. A CD8a+ T Cell Isolation Kit (Miltenyi, 130-104-075) was used for CD8T cell separation according to the manufacturer’s instructions. For spleen or tumor CD8T cell isolation, tissue homogenization was performed first. Red Blood Cell Lysis Buffer (Beyotime Biotechnology, C3702) was then used to lyse the red blood cells, followed by CD8T cell purification. Purified CD8T cells were identified by flow cytometry as >90% isolated cells with CD8^+^ phenotypes.

### Enzyme-linked immunosorbent assay (ELISA)

The ELISA assays used 50 μl of culture supernatant after centrifugation at 16,000 *g* at 4°C for 10 min. The mouse cytokines IL-12 (Biolegend 431704), Il-15 (Biolegend 435104), and IL-18 (Elabscience) were used according to the manufacturers’ instructions. Mouse IL-10 ELISA (Biolegend, 431414), Mouse TNFα ELISA (Biolegend, 430901), and Mouse IL-12p70 ELISA (Biolegend, 433604) were used for intratumor cytokine analysis. For the THP-1 supernatant experiment, Human IL-10 ELISA (Biolegend, 430604) and human IL-6 ELISA (Biolegend, 430504) were used for macrophage polarization analysis. The chemiluminescent signals were measured at 450 nm.

### Quantitative PCR (qPCR)

Total RNA was extracted using a Total RNA Extraction Kit (Solarbio, R1200) according to the manufacturer’s instructions. cDNA was synthesized using the TransScript^®^ Uni All-in-One First-Strand cDNA Synthesis mix (TransGen Biotech, AU341-02). Quantitative PCR (qPCR) was performed using a qPCR Mix (Takara). The relative expression levels of genes were determined using the 2^−ΔΔCt^ method and normalized to h β-actin expression levels. The gene-specific PCR primers are listed below:

*IL6*: F: ACT​CAC​CTC​TTC​AGA​ACG​AAT​TG; R:CCATCTTTGGAAGGTTCAGGTTG;
*TNF*: F: CCT​CTC​TCT​AAT​CAG​CCC​TCT​G; R: GAG​GAC​CTG​GGA​GTA​GAT​GAG;
*IL10*: F: GAC​TTT​AAG​GGT​TAC​CTG​GGT​TG; R: TCA​CAT​GCG​CCT​TGA​TGT​CTG;
*MRC1*: F: TCC​GGG​TGC​TGT​TCT​CCT​A; R: CCA​GTC​TGT​TTT​TGA​TGG​CAC​T;
h β-actin, F: ACT​CTT​CCA​GCC​TTC​CTT​CC; R: CGT​ACA​GGT​CTT​TGC​GGA​TG;

### Statistical analysis

Data were analyzed using GraphPad 9.0 software. Student’s *t*-tests or one-way ANOVA were used to analyze the significance of differences between groups. *P* < 0.05 was considered statistically significant. ****p < 0.0001, ***p < 0.001; **p < 0.01; *p < 0.05.

## Results

### Resveratrol-βcd induces lung adenocarcinoma cell apoptosis and senescence *in vitro*

Due to the low solubility of resveratrol in water, its bioavailability is quite low. Previous studies have reported that inclusion complexes formed by resveratrol-loaded glutathione responsive cyclodextrin nanosponges can increase the water solubility of resveratrol (Palminteri et al., 2021), Therefore, this study used a β-cyclodextrin-resveratrol inclusion complex to improve the resveratrol solubility. The water solubility of resveratrol-β-cyclodextrin (resveratrol-βcd) increased from 0.026 mg/ml to 0.967 mg/ml, with a rapid dissolution rate (Supplementary Figure S2).

**FIGURE 1:**
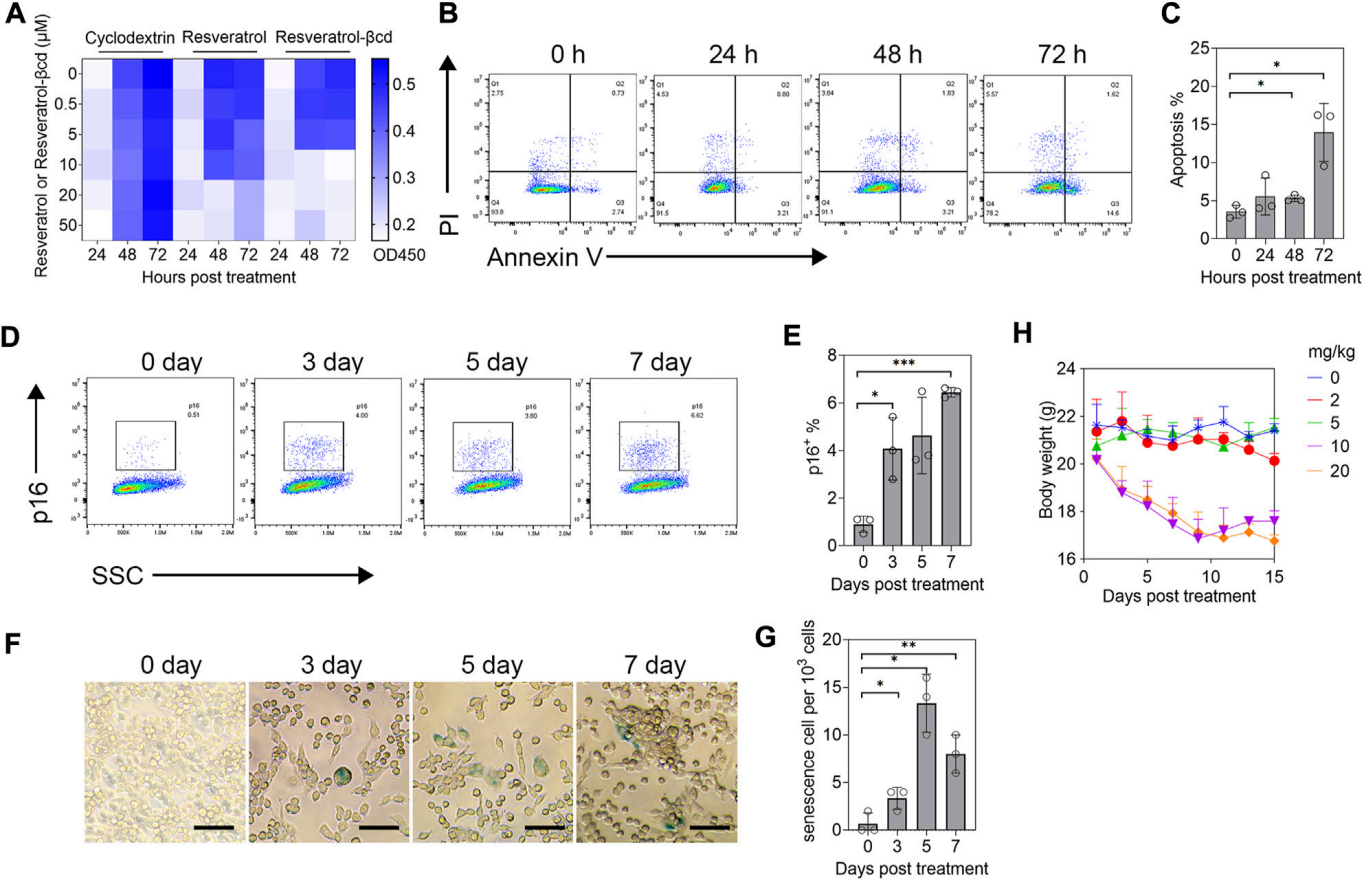
Evaluation of resveratrol-βcd anti-tumor effects *in vitro*. **(A)** CCK-8 assessment of the inhibitory effects of resveratrol-βcd or resveratrol or an equal volume of cyclodextrin on cell proliferation at the indicated times and concentrations. The heatmap values are the average values of three replicates. **(B)** Typical diagram of apoptosis flow cytometry after treatment of LLC cells with 10 μM resveratrol-βcd at different time points. **(C)** Statistical results of apoptosis at the indicated times. Error bar = mean ± S.D. **(D)** Typical flow cytometry diagram of LLC cell senescence analysis after treatment with 10 μM resveratrol-βcd for the indicated times. p16 was used as the marker of senescence. **(E)** Statistical results of the senescence analysis. Error bar = mean ± S.D. **(F)** Typical β-gal graph of LLC cells after treatment with 10 μM resveratrol-βcd at the indicated times. Cyan-stained cells were considered positive for senescence. Scale bar = 50 μm; positive cells per 10^3^ cells were counted. **(G)** Statistical results. Error bar = mean ± S.D. **(H)** Time course of mouse body weight after treatment with the indicated concentrations of resveratrol-βcd. Error bar = mean ± S.D., *n* = 5.

As reported previously, resveratrol can induce tumor cell apoptosis or senescence. Therefore, we first analyzed the therapeutic mechanism of resveratrol-βcd on tumor cells *in vitro*. First, we treated LLC (a lung adenocarcinoma cell line) with 0-50 μM resveratrol-βcd *in vitro* and analyzed cell proliferation at 24, 48, and 72 h post-treatment. The results showed that resveratrol-βcd inhibited tumor growth at 10 μM (Figure 1A). Analysis of apoptosis and senescence after resveratrol-βcd treatment showed that only about 6% of the cells showed apoptosis (both early and late stages) 48 h after treatment (Figures 1B,C). At 72 h after treatment, the level of apoptosis reached approximately 15%. We further analyzed cell senescence by flow cytometry using p16 as a maker of senescence. At 7 days post-treatment, the LLC cells had developed a senescence phenotype (Figures 1D,E). The β-gal analysis showed a similar trend in cell senescence as that observed in flow cytometry analysis (Figures 1F,G). However, the β-gal-positive cell ratio was quite low compared to the p16^+^ ratio. Thus, resveratrol-βcd showed a modest effect on cell senescence. To minimize the effects of the drug on the mice, we evaluated the side effects of different doses of resveratrol-βcd *in vivo*. Subcutaneous injections of resveratrol-βcd were administered every 2 days. At a concentration of 10 mg/kg, the body weight of mice decreased by about 10%, and reduced liver and renal function were observed (Figure 1H, Supplementary Figure S3).

### Resveratrol-βcd activated tumor-infiltrating CD8T cells significantly inhibited subcutaneous tumor growth

**FIGURE 2:**
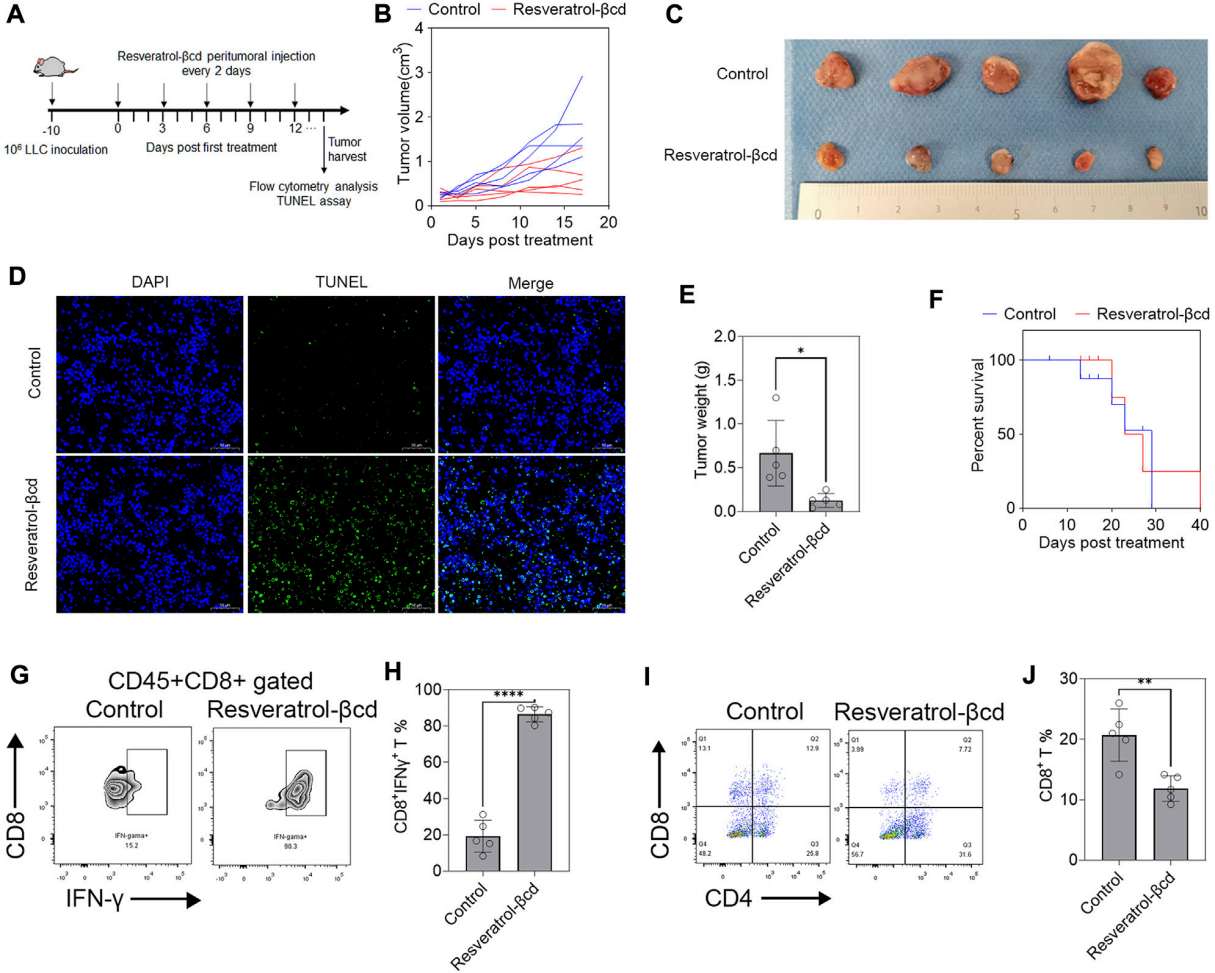
Resveratrol-βcd activates tumor-infiltrating CD8T cells against lung adenocarcinoma. **(A)** A graphical representation showing the timeline of *in vivo* experiments. A total of 10^6^ LLC cells were inoculated into the right flank of mice. Approximately 10 days post-inoculation, the tumor volume reached around 300 mm^3^ and drug administration began. The first day of drug administration was recorded as Day 0 post-treatment. Paratumoral injections were administered every 2 days until reaching the humane endpoint. **(B)** Resveratrol-βcd, equivalent to 5 mg/kg resveratrol, was injected adjacent to the tumor every 2 days, while the control group received the same amount of βcd. The results showed significant inhibition of tumor growth in the treatment group (*n* = 5). **(C)** Typical images of tumors after the mice were sacrificed 17 days after treatment. **(D)** A typical graph of TUNEL analysis of tumor apoptosis 17 days after treatment. Scale bar = 50 μm. **(E)** The mean tumor masses after dissection were 0.126 g and 0.666 g in the treatment and control groups, respectively. Error bar = mean ± S.D., *n* = 5; *p = 0.014. **(F)** Survival curves of the resveratrol-βcd and control groups. All mice in the control group reached the humane endpoint at 29 days post-treatment, compared to 40 days in the resveratrol-βcd group. *n* = 5. **(G)** Typical flow cytometry diagram of CD8T activation in tumors. The cells were CD45^+^CD8^+^ gated. **(H)** CD8T cell activation in the treatment and control groups. Error bar = mean ± S.D., ****p < 0.0001. **(I)** Typical flow cytometry diagram of CD4T and CD8T cells in tumors. The cells were CD45^+^ gated. **(J)** The ratio of CD8T in the treatment and control groups. Error bar = mean ± S.D., **p < 0.01.

We used a murine subcutaneous lung adenocarcinoma model to evaluate the therapeutic effects of resveratrol-βcd (Figure 2A). As shown in Figure 1H, we chose 5 mg/kg resveratrol-βcd for tumor treatment and performed peritumoral injections every 2 days. Compared to the control group, the mice in the treatment group showed obvious tumor inhibition (Figure 2B). On the 17th day after treatment, we sacrificed the mice (Figure 2C). A TUNEL analysis showed increased apoptosis in the treatment group (Figure 2D). In the group administered resveratrol-βcd, the tumor mass was 82% lower (Figure 2E). However, this inhibitory effect only lasted for approximately 10–14 days. In the survival experiment, the resveratrol-βcd-treated mice reached the humane endpoint at about 40 days, about 10 days longer than that in the control group (Figure 2F). Our analysis of the immune cells in the tumor environment on the 17th day after treatment showed that almost all CD8T cells in the tumor were activated in the treatment group (Figures 2G,H); however, the ratio of CD8T cells was significantly decreased (Figures 2I,J). These results suggested that resveratrol may have inhibited tumor growth by activating tumor-infiltrating CD8T cells.

### Tumor-associated macrophages showed a polarization switch during resveratrol-βcd treatment

**FIGURE 3:**
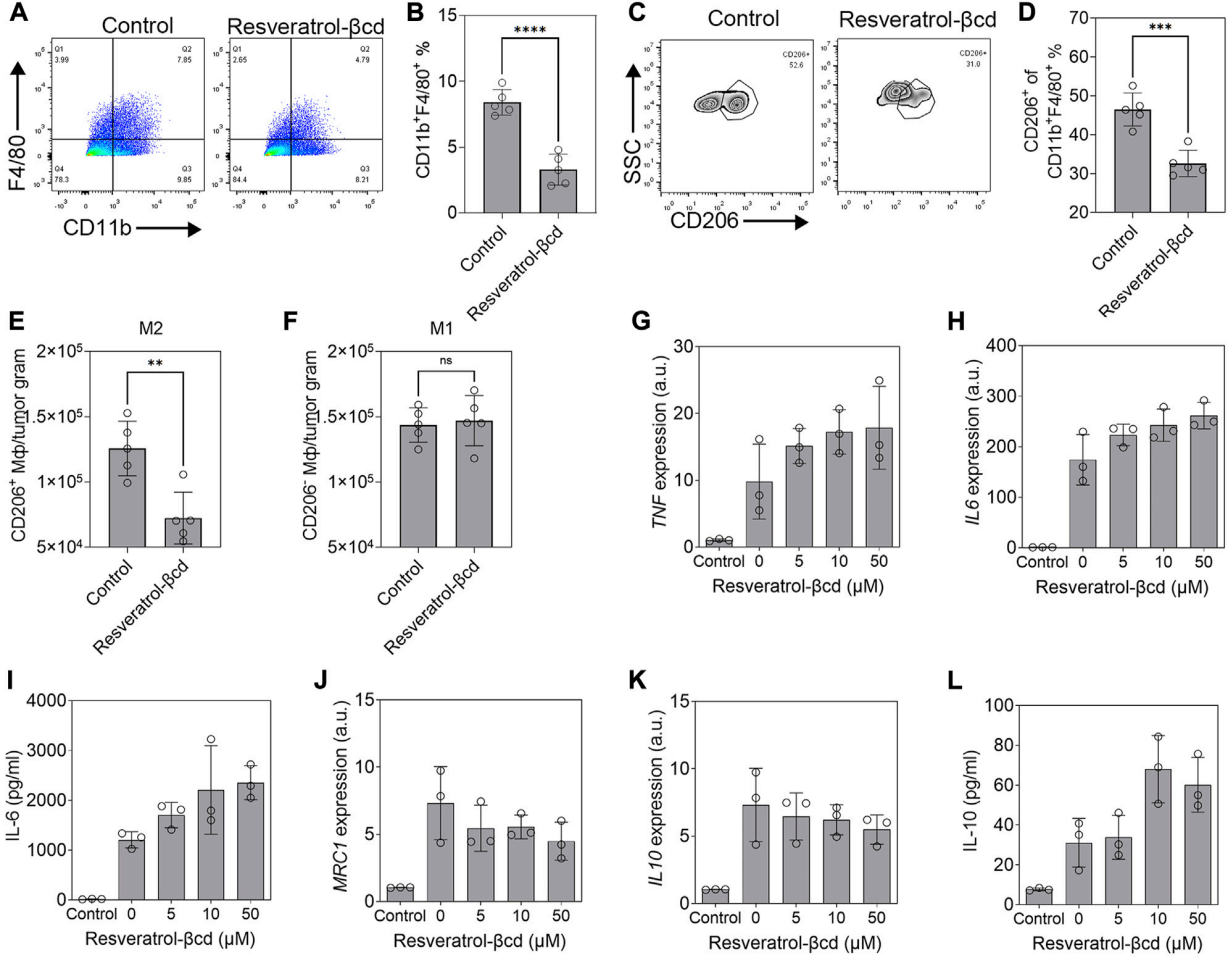
Resveratrol-βcd switch tumor-associated macrophage M2-type polarization. **(A,C)** Typical flow cytometry diagram of TAM (CD11b^+^F4/80^+^) or M2 macrophages (CD11b^+^F4/80^+^ CD206^+^). The cells were CD45^+^ gated. **(B,D)** The proportions of TAM or M2-like macrophages in the treatment and control groups. Error bar = mean ± S.D. ***p < 0.001. ****p < 0.0001. **(E)** The numbers of M2-like and **(F)** M1-like (CD206^-^F4/80^+^CD11b^+^) macrophages per tumor gram. **p < 0.01. **(G)** THP1 as the control group and THP1-derived M1 macrophages treated with resveratrol-βcd at the indicated concentrations. *TNF* and **(H)** *IL6* transcript levels and **(I)** IL-6 levels in the medium 48 h after treatment were measured to identify M1 polarization. Error bar = mean ± S.D. **(J)** *MRC1*, **(K)** *IL10*, and **(L)** IL-10 levels 48 h after treatment with the indicated concentration of resveratrol-βCd. Error bar = mean ± S.D.

The levels of tumor-associated macrophages (TAMs) decreased significantly in resveratrol-βcd treated mice (Figures 3A,B). Using CD206 as a marker of M2-like macrophages, a decreased proportion of M2 macrophages was also observed in the treated group (Figures 3C,D). The number of M2-like macrophages decreased by 42.4% in the treatment group compared to that in the control group (Figure 3E), while the numbers of M1-like macrophages were similar in the treatment and control groups (Figure 3F). Previous studies showed that resveratrol can activate M2 macrophages and show anti-inflammatory effects (Liu et al., 2019; Yu et al., 2019; Ma et al., 2020). We used resveratrol-βcd to treat M1 or M2 macrophages derived from THP1 to determine the effects of resveratrol-βcd on macrophage polarization. The results showed that resveratrol-βcd indistinctly but directly activated THP-1 derived M1 macrophages in a dose-dependent manner (Figures 3G,H) and that IL-6 protein and transcription levels show consistent trends after treatment (Figure 3I). After resveratrol-βcd treatment, the activation levels of different typical markers of M2-like macrophages were not consistent (Figures 3J–L). Resveratrol-βcd had almost no effect on *IL10* transcription levels; however, IL-10 levels increased significantly in the supernatant (Figure 3L). *MRC1(CD206)* transcription levels decreased by 34% after treatment with 50 μM resveratrol-βcd (Figure 3K).

### Resveratrol-βcd activates CD8^+^ T cells through macrophage-derived IL-18

**FIGURE 4:**
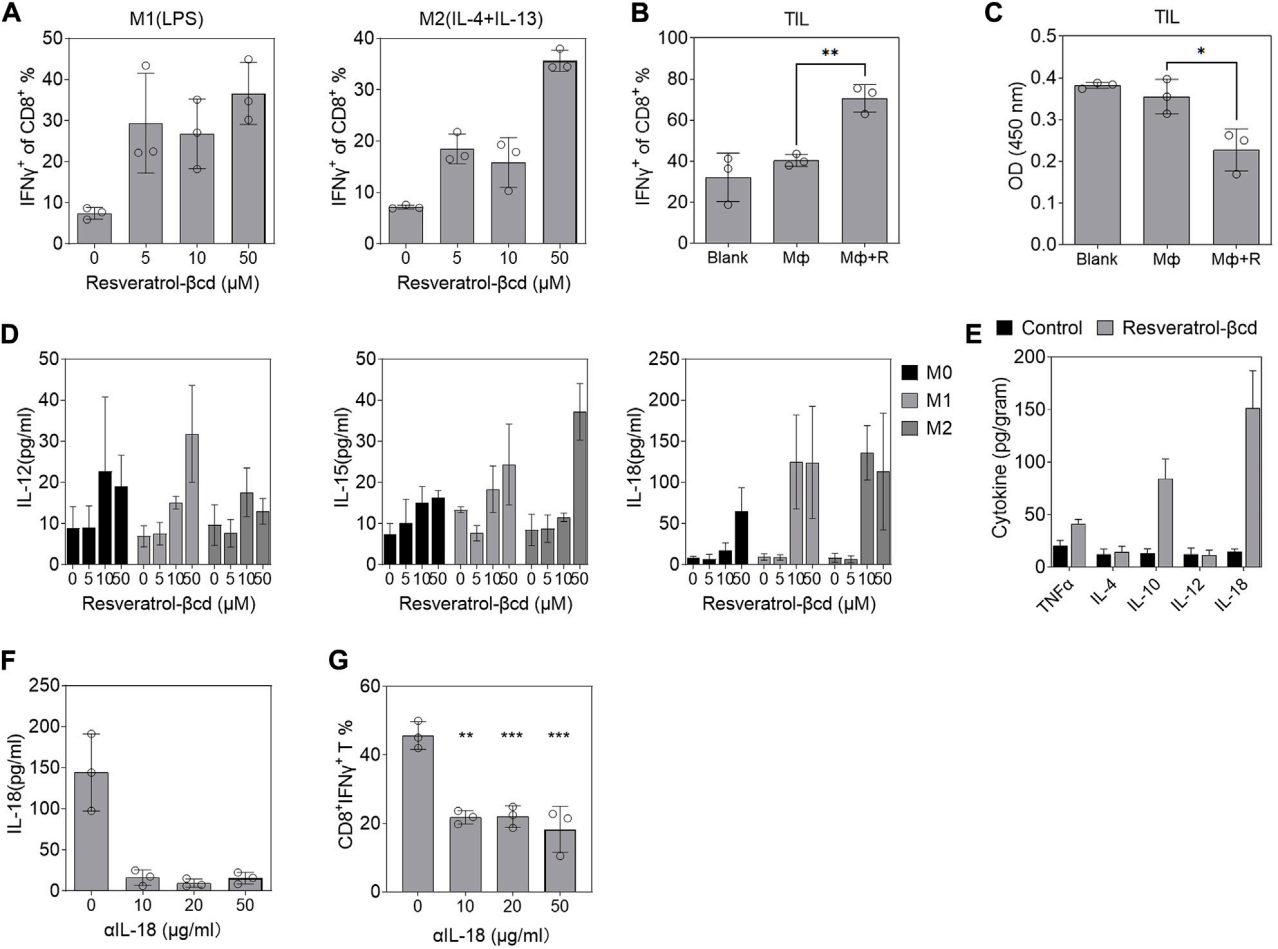
Resveratrol-βcd induced macrophages to secrete IL-18 and promote CD8T bystander activation. **(A)** CD8T activation in co-cultured Raw264.7-derived M1 or M2 macrophages with the indicated resveratrol-βcd and CD8T cells derived from the spleen. Error bar = mean ± S.D. **(B)** Activation of tumor-derived CD8T cells and cell proliferation **(C)** in co-culture with M1 macrophages or M1 macrophages + resveratrol-βcd. Error bar = mean ± S.D. **(D)** M1 or M2 macrophages were activated and incubated with the indicated resveratrol-βcd concentrations for 48 h. The levels of IL-12, IL-15, and IL-18 in the supernatant were analyzed by ELISA. **(E)** Subcutaneous tumors were harvested 4 days after the first treatment. The intratumoral cytokine levels were measured by ELISA. *n* = 5. Error bar = mean ± S.D. **(F)** IL-18 in the supernatant of M1 macrophages was neutralized by the indicated anti-IL-18 antibody (αIL-18) and the effect of neutralization was analyzed. Error bar = mean ± S.D. **(G)** After neutralization, M1 macrophages treated with resveratrol-βcd were co-cultured with spleen-derived CD8Ts. CD8T activation was analyzed 24 h after co-culture. Error bar = mean ± S.D., **p < 0.01, ***p < 0.001.

We co-cultured Raw 264.7-derived M1 (LPS) or M2(IL-4+IL-13) with spleen-derived CD8T cells in different concentrations of resveratrol-βcd and analyzed CD8T cell activation. CD8T cells were partially activated under 5 μM resveratrol-βcd (Figure 4A). Moreover, M1 macrophages elicited significantly higher levels of CD8T activation compared to M2 macrophages (Figure 4A). However, these activation levels (40%) were not consistent with the results observed in the tumor (Figure 2H), which showed >90% activation. This difference might be due to the specificity of tumor-infiltrating CD8T cells. Therefore, we sorted tumor-infiltrating CD8T cells from mice LLC tumors for co-culture. The results showed that tumor-derived CD8T was significantly activated with macrophages and 10 μM resveratrol-βcd (Figure 4B), indicating that tumor-derived CD8T cells had a phenotype that could be activated by macrophages. Interestingly, CD8T cells barely proliferated after co-culture, and the cell population decreased even in culture with resveratrol-βcd (Figure 4C). Our analysis of IL-12, IL-15, and IL-18 levels, which are associated with CD8T activation, in the macrophage culture medium 48 h after resveratrol-βcd treatment showed that IL-12 and IL-15 levels were not significantly increased in M0, M1, or M2 macrophages (Figure 4D), while IL-18 levels were greatly increased in both M1 and M2 macrophages. In the tumors, IL-10 and IL-18 levels also increased 4 days after the first treatment (Figure 4E). An antibody neutralization experiment was performed to clarify the role of macrophage-derived IL-18 in CD8T activation. The addition of 10 μg/ml αIL-18 (anti-IL-18 antibody) to the medium reduced the IL-18 level to approximately 10 pg/ml (Figure 4F) and significantly inhibited CD8T cell activation (Figure 4G). These results suggested that IL-18 is a factor involved in resveratrol-βcd-induced CD8T cell activation through macrophages.

## Discussion

Immune cells are highly abundant in the tumor microenvironment of lung adenocarcinoma. CD8T cells are the most important type of immune cells involved in tumor immunity and have direct cytotoxic effects. Regarding CD8T cell activation, in addition to the classical TCR pathway mediated activation, there also exists a bystander mode of activation. Various types of human cancers show many CD8^+^ T cells without tumor antigen specificity (Simoni et al., 2018). This type of CD8T cell can be activated in a T-cell receptor-independent, cytokine-dependent manner. The cytokines that can participate in the bystander activation of CD8T cells include type I interferon (Tough et al., 1996), interleukin-18 (Freeman et al., 2012), and interleukin-15 (Kim et al., 2018). Bystander-activated CD8T cells are cytotoxic and secrete cytokines such as interferon-γ to perform their effects; however, bystander activation is nonspecific and tends to promote T cell activation rather than proliferation. The results of the present study demonstrated a significant increase in the level of T cell activation after treatment but a significant decrease in the absolute number of T cells. This indicated that resveratrol-βcd may activate T cells through bystander activation. Cytotoxic activity and the production of TNFα and IFN-γ are the main mechanisms of the CD8^+^ T cell-mediated tumor cytotoxic effect. Both IFN-γ and TNF-α can be induced when CD8^+^ T cells are stimulated with peptide antigens. However, during bystander activation, CD8^+^ T cells only produce IFN-γ, rather than TNFα (Beadling and Slifka, 2005). Resveratrol reportedly attenuates the proliferative effect of TNF-α on CD8T cells *in vitro* (Silva et al., 2014), likely because resveratrol can activate and cause the apoptosis of CD8T *via* the bystander pathway.

In addition, macrophages are among the most abundant immune cells in tumors. TAMs usually exhibit M2-like polarization, which is associated with tumor immune escape, angiogenesis, and invasion during tumor development. TAMs mainly play a role in tumor immunosuppression during the development of lung adenocarcinoma. This immunosuppressive effect is partly caused by crosstalk with CD8T cells.TAM can express PD-L1, PD-L2, and CTLA-4 ligands CD80 and CD86. These immune checkpoints mainly target CD8 T cells and lead to the inhibition of CD8T cytotoxic effects. TAM also plays an important role in CD8^+^ T cell infiltration in malignant tumors (Wynn and Barron, 2010; Jiang et al., 2017). *In vivo* studies showed that TAM-derived TGF-β inhibited T cell-mediated tumor clearance (Thomas and Massague, 2005). The neutralization of TGF-β by an antagonist upregulated cytotoxic gene expression in CD8^+^ T cells in a mouse model (Thomas and Massague, 2005). TAM also modulated T cell metabolism (Noy and Pollard, 2014; Proto et al., 2018).

Infectious disease studies reported that resveratrol activates M2 macrophages (Liu et al., 2019; Yu et al., 2019; Ma et al., 2020) and reduces the proportion of M1 macrophages (Shabani et al., 2020; Cheuk et al., 2022). Resveratrol inhibits macrophage infiltration and IL-6 secretion during infection (Li et al., 2020). These findings suggest that resveratrol may alleviate the suppressive state of T cells in the tumor environment by altering macrophage polarization or attenuating macrophage M2 polarization. Resveratrol and its analogs can inhibit hematological tumors *in vitro* (Anisimova et al., 2011). Resveratrol has a concentration-dependent bidirectional function: while it significantly activates NK cells at low concentrations, it shows inhibitory effects at high concentrations (Falchetti et al., 2001). Resveratrol also slightly inhibits the production of interleukin-10 by approximately 10% and monocyte chemoattractant protein-1 production by approximately 50% in M2 macrophages, while promoting the production of transforming growth factor-β1 (Kimura and Sumiyoshi, 2016). The results of the present and previous studies suggest that resveratrol likely inhibits macrophage polarization, reduces cytokine secretion, and indirectly promotes T cell activation.

In conclusion, we used a subcutaneous tumor model of lung adenocarcinoma to evaluate the antitumor effects of resveratrol. The results demonstrated the anti-tumor effects of resveratrol at concentrations that did not affect the normal physiological activity of mice. In addition to its direct inhibitory effect on tumor cell growth, resveratrol also indirectly activated T cells by switching macrophage polarization. Macrophage-derived IL-18 may be a key bystander activation cytokine during resveratrol treatment. During treatment, the proportions and immune state of macrophages and T cells within the tumor environment showed obvious changes, suggesting that resveratrol could switch the tumor immune microenvironment, reverse the immunosuppression state of these immune cells, and activate CD8 T cells, thus providing an anti-tumor effect. The reduced proportion of T cells during treatment suggested further research to assess the combination of resveratrol with immune stimulators such as IL-2 to potentially improve the therapeutic effect.

## Data Availability

The original contributions presented in the study are included in the article/Supplementary Material. Further inquiries can be directed to the corresponding authors.
