# Supplementary material for: Resveratrol activates CD8+ T cells through IL-18 bystander activation in lung adenocarcinoma
Source: Front Pharmacol. 2022 Oct 20;13:1031438. doi: 10.3389/fphar.2022.1031438 (PMC9630476; doi:10.3389/fphar.2022.1031438)
Supplement: Supplementary file 1 [file DataSheet1.DOCX]

Supplementary Material


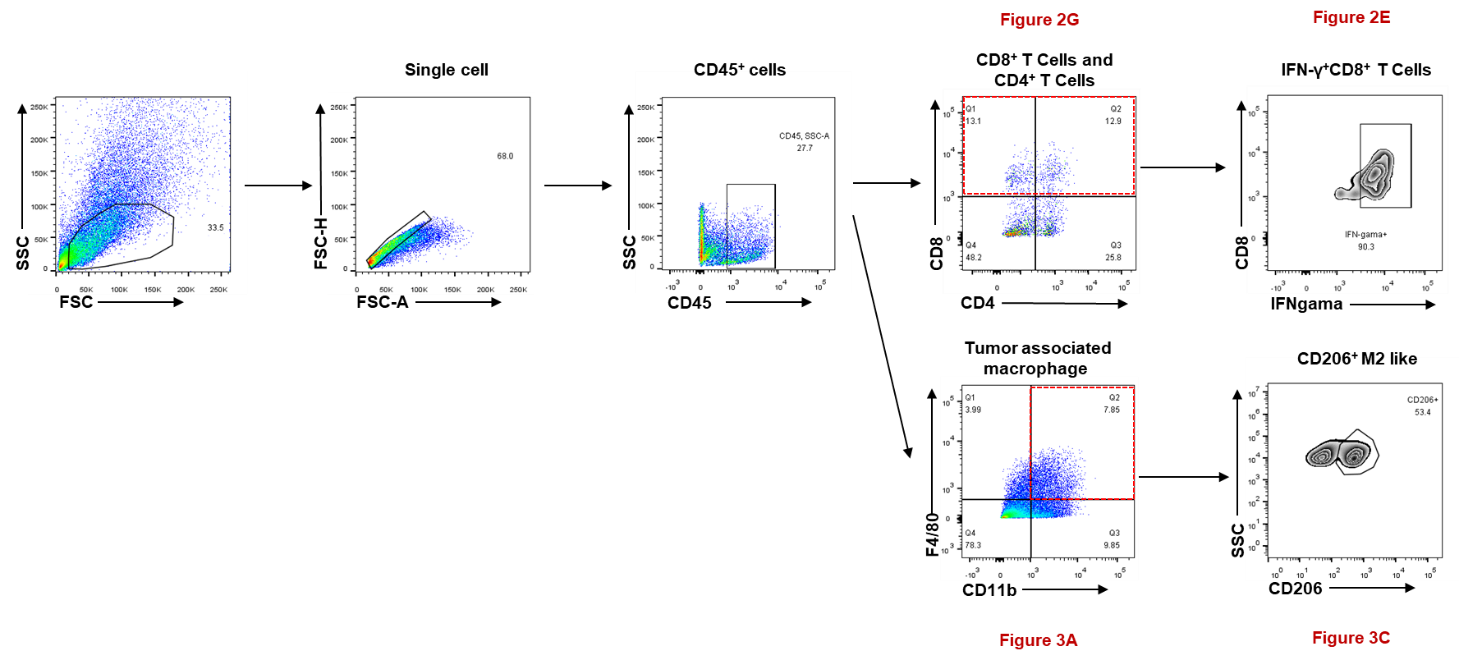


**Supplementary Figure 1.** Exemplifying gating strategies for FACS analysis are shown. Gating strategy for tumor-infiltrating CD8^+^ T cells (for Figure 2G) and its activation (for Figure 2E); gating strategy for tumor-associated macrophages (for Figure 3A), M2-like(CD206^+^) cells (for Figure 3C).
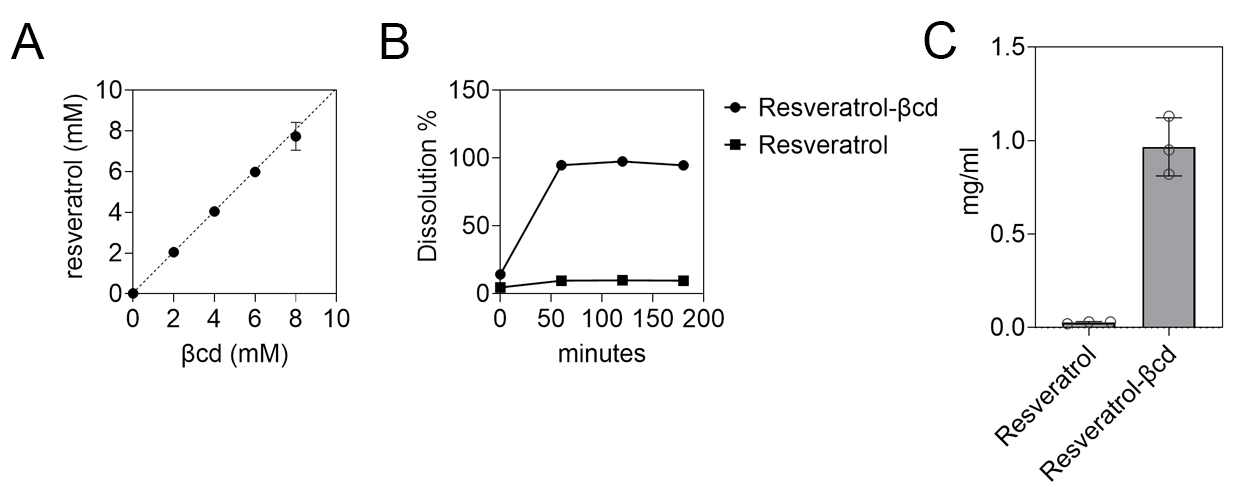


**Supplementary Figure 2.** Water solubility diagram of resveratrol-βcd. **(A)** the complex formed in 1:1 in the range of 0.0–8.0 mM βcd; **(B)** dissolution rate of resveratrol and resveratrol-βcd in water at 37.0 ± 0.1 °C. Error bar=±S.D.;**(C)** the water solubility of resveratrol-βcd increased from 0.02 mg/ml to 0.96 mg/ml,Error bar=±S.D.

**
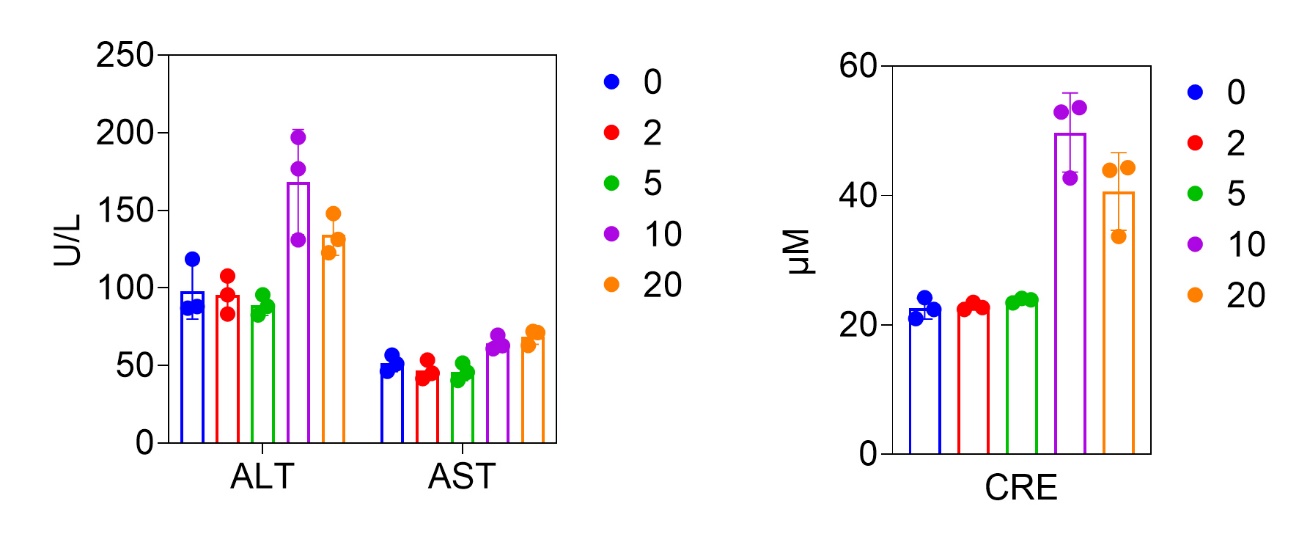
**

**Supplementary Figure 3.** Blood biochemistry of C57BL/6 mice treated with resveratrol-βcd at 7 days. Serum levels of alanine aminotransferase (ALT), alkaline phosphatase (ALP), creatinine (CRE) were determined. Data are shown as the mean ± standard deviation (s.d.) (n = 3 independent experiments).
